# A Missense Variant in *HACE1* Is Associated with Intellectual Disability, Epilepsy, Spasticity, and Psychomotor Impairment in a Pakistani Kindred

**DOI:** 10.3390/genes15050580

**Published:** 2024-05-02

**Authors:** Muhammad A. Usmani, Amama Ghaffar, Mohsin Shahzad, Javed Akram, Aisha I. Majeed, Kausar Malik, Khushbakht Fatima, Asma A. Khan, Zubair M. Ahmed, Sheikh Riazuddin, Saima Riazuddin

**Affiliations:** 1 Department of Otorhinolaryngology Head & Neck Surgery, School of Medicine, University of Maryland, Baltimore, MD 21201, USA; asaadusmani88@gmail.com (M.A.U.); mmghaffar29@gmail.com (A.G.); 2 Department of Molecular Biology, Shaheed Zulfiqar Ali Bhutto Medical University, Islamabad 44000, Pakistan; mohsinzoologist@gmail.com (M.S.); jakramaimc@gmail.com (J.A.); riazuddin@aimrc.org (S.R.); 3 Jinnah Burn and Reconstructive Surgery Center, Allama Iqbal Medical College, University of Health Sciences, Lahore 54550, Pakistan; 4 Center of Excellence in Molecular Biology, University of the Punjab, Lahore 54500, Pakistan; 5 Department of Radiology, Shaheed Zulfiqar Ali Bhutto Medical University, Islamabad 44000, Pakistan; ayeshamajeed1@gmail.com; 6 Department of Applied Health Sciences, University of Management and Technology, Lahore 54500, Pakistan; khushbakht.fatima98@gmail.com; 7 Department of Molecular Biology and Biochemistry, School of Medicine, University of Maryland, Baltimore, MD 21201, USA

**Keywords:** intellectual disability, NDD, epilepsy, autosomal recessive, exome sequencing

## Abstract

Intellectual disability (ID), which affects around 2% to 3% of the population, accounts for 0.63% of the overall prevalence of neurodevelopmental disorders (NDD). ID is characterized by limitations in a person’s intellectual and adaptive functioning, and is caused by pathogenic variants in more than 1000 genes. Here, we report a rare missense variant (c.350T>C; p.(Leu117Ser)) in *HACE1* segregating with NDD syndrome with clinical features including ID, epilepsy, spasticity, global developmental delay, and psychomotor impairment in two siblings of a consanguineous Pakistani kindred. *HACE1* encodes a HECT domain and ankyrin repeat containing E3 ubiquitin protein ligase 1 (HACE1), which is involved in protein ubiquitination, localization, and cell division. HACE1 is also predicted to interact with several proteins that have been previously implicated in the ID phenotype in humans. The p.(Leu117Ser) variant replaces an evolutionarily conserved residue of HACE1 and is predicted to be deleterious by various in silico algorithms. Previously, eleven protein truncating variants of HACE1 have been reported in individuals with NDD. However, to our knowledge, p.(Leu117Ser) is the second missense variant in HACE1 found in an individual with NDD.

## 1. Introduction

Neurodevelopmental disorders (NDD) with their phenotypic diversities show a 5% to 20% global prevalence, while intellectual disability (ID) with a global percentile of 2% to 3% prevails at 0.63% of the overall NDD prevalence [1,2]. However, a larger prevalence is reported in less-developed countries and populations with a high frequency of consanguineous marriages. The etiology of ID has been equally distributed among environmental factors and genetic deficits [3]. It has been estimated that variants in around 2000–3000 known human genes might be implicated in the genetic foundation of ID, many of which have yet to be identified [4,5].

In this study, through exome sequencing, we identified a rare variant in the *HACE1* gene co-segregating with the NDD phenotype in a Pakistani family showing a recessive pattern of inheritance. HACE1 consists of two major structured domains including six ankyrin repeats at the amino terminus, responsible for protein–protein interactions and binding to the ligase targets, and a carboxy terminus HECT domain responsible for E3 ubiquitin–protein ligase activity [6]. HACE1 acts as an E3 ubiquitin ligase and interacts with the E2 enzyme UBCH7 to tag proteins for ubiquitination by the 26S proteosome [7,8]. Murine-level studies show a high expression of HACE1 in brain tissues in contrast to other organs.

**Figure 1:**
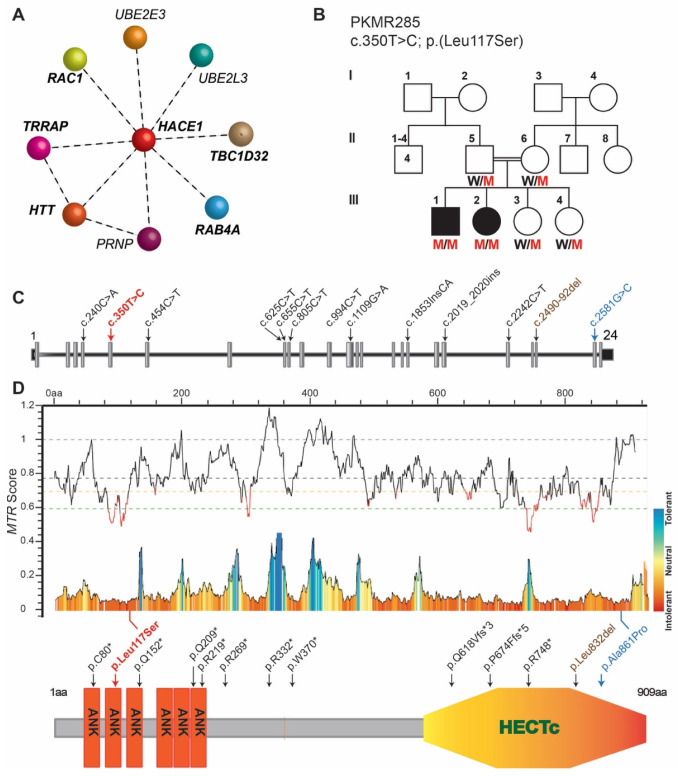
A novel missense variant in *HACE1* segregates in PKMR285. (**A**) STRING analysis based on the HACE1 interactome, which includes proteins, TRRAP, TBC1D32, HTT, RAC1, RAB4A, and PRNP, previously implicated in ID-disorder in humans. (**B**) Family structure of PKMR285. Consanguineous marriage is shown by double horizontal line, filled symbols represent affected individuals, and genotypes for the identified *HACE1* variant are given for the participating individuals. W: wild type allele; M: mutant allele. (**C**) Structure of *HACE1* gene, along with all the known variants leading to frameshift (black), in-frame del (brown), and a missense substitution (blue), along with the novel variant c.350T>C (red) found in the current study. (**D**) Tolerance landscape visualization of *HACE1* via MetaDome with relative positions of all reported variants along with protein structure and position of identified variants within HACE1.

HACE1 is known to interact with Rac1 (Figure 1A), a well-known member of the Rho GTPase family, which participates in cerebellar development by controlling processes like cell morphogenesis and migration [6,9]. HACE1 controls oxidative stress at the cellular level through the ubiquitination of Rac1 and Nrf2. Higher cellular levels of Rac1 can result in the production of excessive reactive oxygen species (ROS). HACE1 ubiquitination regulates Rac1 activity, resulting in the reduction in ROS, and mitochondrial dysfunction, hence maintaining cellular hemostasis. Nrf2 is a well-known transcription factor responsible for gene activation in response to stress, and HACE1 regulates the translation of Nrf2 and its stability [10]. HACE1 also controls neuronal differentiation, regeneration, and neurite growth through the transcriptional regulation of RARα1, RARβ:1, RARβ:2, and RARβ:3 of the CRABPII/RAR pathway [11,12,13]. Optineurin (OPTN), an autophagic receptor, is also a known HACE1 substrate, which interacts with Huntington protein and protects neurons from damage. STRING analysis predicts the interaction of HACE1 with several proteins known to be implicated in human neurodevelopmental disorders (Figure 1A). In summary, HACE1 has direct or indirect involvement in neuronal protection against oxidative stress, mitochondrial dysfunction, autophagic dysfunction, and neuroinflammation that can cause neurodegeneration disorders [10]. In humans, *HACE1* variants have been reported in individuals with spastic paraplegia and psychomotor retardation with or without seizures [8,14,15,16]. To date, ten protein truncating, one in-frame deletion, and one missense variants in *HACE1* have been reported in subjects with neurological disorders [8,14,15], and our study represents the second known case of *HACE1* disorder associated with a novel missense allele.

## 2. Material and Methods

### 2.1. Family Enrolment

This study was approved by the Institutional Review Board (IRB) of the Centre of Excellence in Molecular Biology (CEMB), Lahore, Shaheed Zulfiqar Ali Bhutto Medical University (SZABMU), Islamabad, Pakistan, and University of Maryland Baltimore (UMB; HP-00075913; renewed on 1 May 2024), USA. After written informed consent, Family PKMR285 was ascertained from Khyber Pakhtunkhwa Province of Pakistan. Peripheral blood samples were collected from all the participating individuals for genomic DNA extraction. Clinical assessment was conducted on each individual to determine presence of intellectual disability, vision or hearing impairment, ataxia, and bone deformities.

### 2.2. Exome and Sanger Sequencing

Exome sequencing (ES) was carried out using an Illumina HiSeq4000 system (Illumina, San Diego, CA, USA) with average 100× coverage, and data analysis was performed as described previously [3]. Briefly, the Burrows Wheeler aligner (BWA) was used to align the reads, while variant calling was performed through Broad Institute’s Genome Analysis Toolkit (GATK) (https://gatk.broadinstitute.org/hc/en-us (accessed on 14 January 2021)). Candidate variants with CADD scores ≥ 20 and an allele frequency of ≤0.001% in gnomAD, 1000 Genomes, and NHLBI ESP were filtered, prioritized, and analyzed for segregation using the Sanger sequencing technique.

### 2.3. In-Silico Analysis and 3D Protein Modelling

Various online available pathogenicity analysis tools, including Varsome, Marrvel, MutationTaster, Polyphen-2, PhyloP, SIFT, FATHMM, and GERP++, were used to assess the pathogenicity scores of the *HACE1* identified variant. Clustal Omega was used for evolutionary conservation of mutated residues, while MetaDome interface helped to assess the missense intolerance scores. Further, Pymol, a molecular visualization system, and the HOPE tool were used to perform the 3D modelling of the wild type and mutated residues. We also analyzed the single-cell RNA (sc-RNA) expression of HACE1 along with other important proteins for amino acid synthesis and interconversion using the UCSC cell browser database for developing telencephalon.

## 3. Results

### 3.1. Family PKMR285

Family PKMR285 was ascertained from Khyber Pakhtunkhwa Province of Pakistan, and has two affected siblings, a 23-year-old male and a 25-year-old female (Figure 1B). Both siblings have a clinical presentation of severe intellectual disability, epilepsy, spasticity, and psychomotor impairment along with global developmental delay (NDD) with no major facial dysmorphism.

### 3.2. Genetic Studies

To determine the underlying cause of the NDD phenotype, we subjected a DNA sample of proband (III:1) to exome sequencing. Identified genomic variants were analyzed through the multi-tier filtration process as previously described [3], which revealed four candidate variants, including three missense and one splice-site variant. All these four variants were Sanger-sequenced in the DNA samples of participating family members, which revealed the co-segregation of a homozygous novel variant, c.350T>C in exon 5 of the *HACE1* gene, with the NDD phenotype in Family PKMR285 (Figure 1B,C). The c.350T>C variant is predicted to replace a leucine residue at amino acid position 117 with serine (p.Leu117Ser). Previous studies have reported ten frameshift, one in-frame deletion, and one missense variant in HACE1 (Figure 1C). The variant p.(Leu117Ser) represents the second known missense variant of HACE1 associated with NDD in humans.

The missense tolerance ratio (MTR), a measure of the regional intolerance to missense variation, underwent analysis and a score of 0.689 was calculated for the p.Leu117Ser variant, which was consistent with the location of the p.Leu117 residue within the highly intolerant region of the ANK domain of HACE1 (Figure 1D). Moreover, several in silico prediction algorithms supported the deleterious or damaging impact of the identified variant, similar to other known HACE1 variants (Table 1).

**Figure 2:**
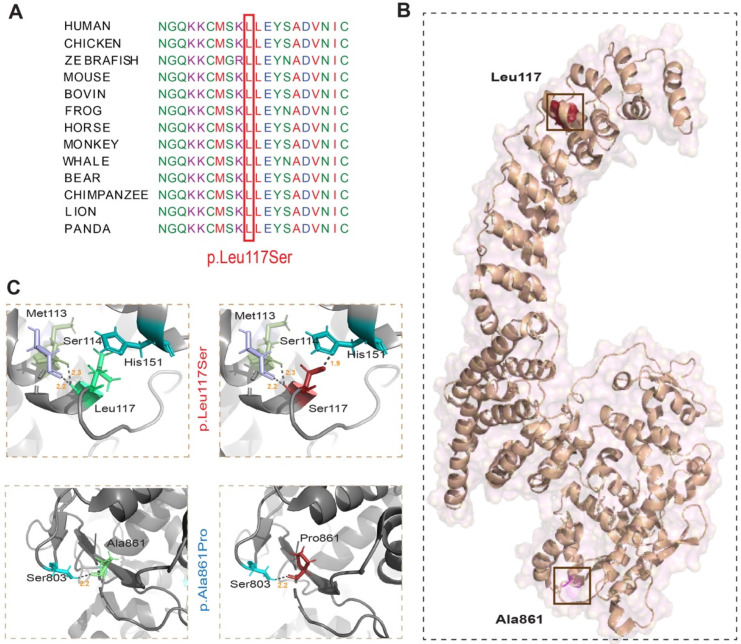
Evolutionary conservation, and 3D protein modeling of missense variants identified in HACE1. (**A**) Phylogenetic analysis showed that the p.Leu117 residue of HACE1, mutated in Family PKMR285, is evolutionarily conserved in vertebrates. (**B**) 3D protein modeling of HACE1. (**C**) Zoomed position of wild-type and p.Leu117Ser (novel) and p.Ala861Pro (known) variants harboring proteins. Hydrogen bonding between the residues is shown with dotted lines along with the distances in Å. Substitution of p.Leu117 with p.Ser117 is predicted to cause a new interaction with p.His151 through a strong polar bond with a distance of 1.9 Å. In contrast, the p.Ala861Pro substitution is not predicted to affect the interaction with other amino acids or generate new interactions.

Phylogenetic analysis showed that the p.Leu117 residue is evolutionarily conserved (Figure 2A). To gain further insights on the potential impact of the identified variant on the protein secondary structure, we performed three-dimensional (3D) protein modelling (Figure 2B). We also included the previously reported p.Ala861Pro variant in our in silico analysis (Figure 2B). For 3D protein modeling, we used the human HACE1 protein structure PDB: 8H8X and Pymol program. The p.Leu117 residue is predicted to form two hydrogen bonds with p.Met113 (bond length 2.2 Å) and p.Ser114 (2.3 Å; Figure 2C). Substitution of p.Leu117 with p.Ser117 is predicted to result in a new aberrant interaction with histidine at position 151 (Figure 2C). Moreover, the small size of serine and its lower hydrophobicity as compared to the wild-type leucine residue is predicted to cause the loss of hydrophobic interactions and the presence of empty space in the core of the protein. In contrast, the p.Ala861 residue shares a hydrogen bond with p.Ser802 (2.2 Å), which is not altered due to the p.Ala861Pro variant (Figure 2C). However, the mutant proline residue is bigger in size, and the residue is located on the surface of the HECT domain of HACE1, and thus could impact the interactions with other binding partners.

**Figure 3:**
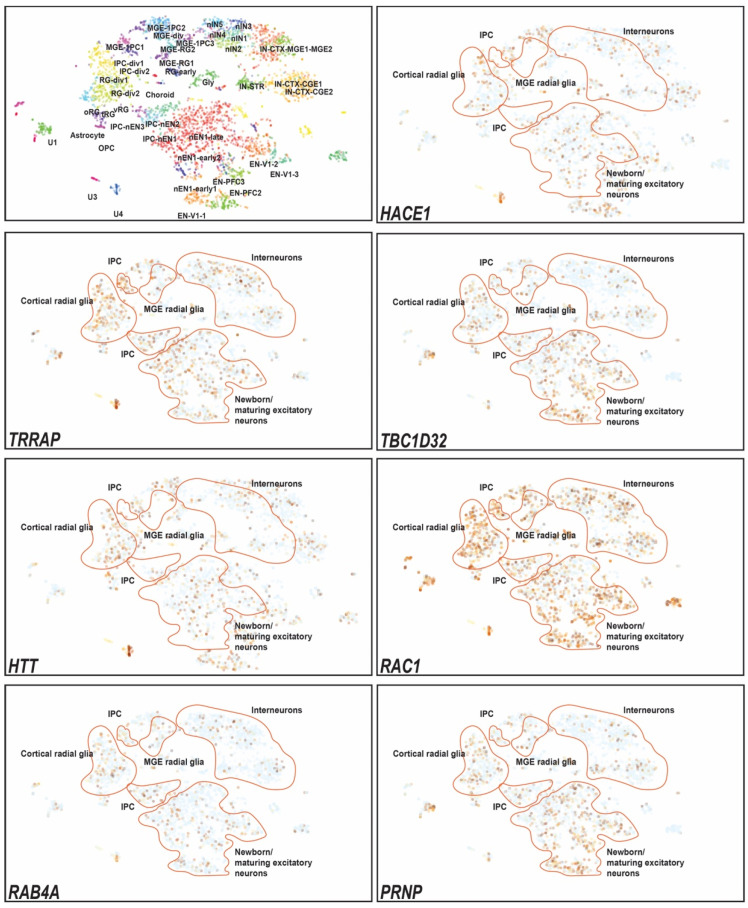
Single-cell RNA expression analysis of HACE1 and its interactor genes, already known to cause ID, in developing human brain tissues. Single-cell RNA-seq (sc-RNA seq) visualization of *HACE1* in human developing telencephalon. Data were obtained from the UCSC cell browser for cortex development dataset, generated from the expression of 4261 cells. Gene panels show expression data plotted in t-SNE on the WGCNA layout, areas of interest are highlighted with orange lines, beige to dark brown show high RNA expression levels, whereas blue shows the absence of expression. For cell type clustering details, see https://cells.ucsc.edu/?ds=cortex-dev (accessed on 16 December 2023). MGE: medial ganglionic eminence. IPC: intermediate progenitor cells. RNA expression analysis shows the overlap of *HACE1* with its known and predicted interactors, which are known to cause ID, including *TRRAP*, *TBC1D32*, *HTT*, *RAC1*, *RAB4A*, and *PRNP.* Expression seemed to be highly overlapping in cortical radial glia, excitatory neurons, and IPC.

Next, we used publicly available databases of developing human telencephalon to analyze *HACE1* and its known and predicted binding partners’ expression. High overlapping expression of *HACE1* with these interacting partners was observed in the maturing excitatory neuron clusters, intermediate progenitor cells, and radial glia cells (Figure 3).

## 4. Discussion

In this study, we identified and report the first known variant of *HACE1* in a Pakistani family, segregating with the NDD phenotype. Previously, twelve variants of *HACE1* have been reported in twenty individuals from various ethnicities. All these subjects share similar phenotypes including intellectual disability, cephalic abnormalities, hypotonia, spastic paraplegia, mute or limited speech, and psychomotor impairment with or without seizures [8,14,15]. Similarly, the two affected individuals of PKMR285 also have intellectual disability, seizures, verbal limitation, spasticity, and psychomotor impairment. However, the current HACE1 variants’ harboring cohort size is not large enough for meaningful genotype–phenotype correlation studies.

HACE1 exhibits dual functionality as an E3 ligase, capable of facilitating the degradation process through two distinct mechanisms that vary in their reliance on E3 ligase activity [10]. HACE1, through its HECT domain E3 ligase activity, is involved in proteasomal degradation, while its ANK domain-mediated protein–protein interactions contribute to autophagic degradation [10]. Both domains seem to be hotspot regions for identified variants, as 10 of the known 13 variants, including the variant found in Family PKMR285, lay in these structured regions (Figure 1D). The overexpression of constructs lacking ANK repeat regions in MEFs derived from the *Hace1*^−/−^ mouse model treated with puromycin fail to localize with protein aggregates and lost the ability to bind and deliver target proteins for autophagic degradation, hence increasing the cellular toxicity leading to cell death [17], and thus highlighting the crucial role of ANK domains in HACE1 function.

In the brain, scRNA data analysis revealed the higher expression overlap of *HACE1* with *TRRAP*, *TBC1D32*, *HTT*, *RAC1*, *RAB4A*, and *PRNP* in cortical glia, excitatory neurons, and intermediate progenitor cells. TRRAP is a transformation/transcription domain-associated protein kinase with epigenetic-based transcription activity and acts as a checkpoint in cell division to control chromatin remodeling and repair DNA breaks [18], while TBC1D32 is known to participate in the Hedgehog signaling pathway and regulates the structure of the primary cilium in the neural tube [19]. The HTT huntingtin protein involve in neural development has been shown to dysregulate cell migration, reduce proliferation, and increase cell death in the neocortex of murine *Htt* knockouts [20,21]. Higher RAC1 expression has been reported to increase ROS content and CD1 expression, hence increasing the mTOR stability which ultimately leads to cell death [22,23]. In the neurons, RAC1 dysregulation results in reduced numbers of synapses, and spines for dendrites [23]. RAB GTPases including RAB4A are involved with vesicle trafficking; specifically in the brain, RAB4A is involved in neuronal transport through AMPA (α-amino-3-hydroxy-5-methyl-4-isoxazolepropionic acid receptor) receptor subunits and hence in the reverse control synapse [24]. PRNP (prion protein) mutant mice show undifferentiated oligodendrocytes and delayed expression of differentiation markers however proliferation increases [25]. Thus, the overlapping expression and binding with some of these proteins might implicate HACE1 in regulating their spatiotemporal expression profile to ensure normal neuronal differentiation, regeneration, neurite growth, and synapse [16].

HACE1 shows a neuroprotective role against damage during oxidative stress, mitochondrial and autophagic dysfunction, and neuroinflammation. During oxidative stress, HACE1 helps in the higher transcription and stabilization of Nrf2 and increased degradation of Rac1. Rac1 has the capability to form a complex with PI 3-Kinase, P85α, and HTT during oxidative stress, which form protein aggregates, hence damaging neuronal cells. Ubiquitination through HACE1 leads to the degradation of Rac1, and thus prevents cellular damage from protein aggregates. HACE1, along with UPS (ubiquitin proteasome system) proteins, also shows anti-inflammatory effects through the downregulation of the IRF3 (Interferon regulatory factor 3) and NF-κB activation pathways. The transcriptional factors NF-κB and IRF3 further induce the expression of pro-inflammatory cytokines and type I IFN involved in the immune response. HACE1, along with UPS proteins, shows an anti-inflammatory effect by downregulating the IRF3 and NF-κB activation pathways, hence showing the involvement of UPS and HACE1 in immune-related pathogenesis and neurodevelopmental disorders [26]. In summary, HACE1 participates in several different pathways that directly or indirectly regulate neuronal development and cellular signaling, and the pathogenic variants of HACE1 identified in human subjects likely cause both neurodevelopmental as well as neurodegenerative disorders through the dysregulation of these signaling pathways in the brain.

## Figures and Tables

**Table 1:** In silico analysis of identified pathogenic variants in *HACE1*.

Family	PKMR285	Family 4	Family 1 [14]
**Origin**	**Pakistan**	**United Kingdom**	**Turkish**
**Affected Individuals**	**2 Siblings**	**2 Siblings**	**3 Siblings**
**Inheritance**	Homozygous	Homozygous	Homozygous
**hg19 Coordinates**	6:105291150 A>G	6:105178224 C>G	6:105192058-60del AAG
**Nucleotide Variant**	c.350 T>C	c.2581 G>C	c.2494-96del CTT
**Amino Acid Substitution**	p.Leu117Ser	p.Ala861Pro	p.Leu832del
**Nucleotide Reference**	NM_020771.4		
**ACMG Classification**	Pathogenic Strong	Pathogenic Moderate	Pathogenic
**ACMG Criteria**	PP3	PP3	NA
**Meta Score ^a^**	15		NA
**gnomAD Frequencies**	Zero	Zero	NA
**DANN**	Uncertain	Uncertain	NA
**CADD**	26.8	24.5	NA
**REVEL**	Pathogenic Moderate	Benign Moderate	NA
**M-CAP**	Damaging	Tolerated	NA
**DOGEN2**	Benign Supporting	Benign Moderate	NA
**PROVEAN**	Pathogenic Supporting	Benign Moderate	NA
**Polyphen-2 HumDiv**	Probably Damaging	Probably Damaging	NA
**Polyphen-2 HumVar**	Probably Damaging	Possibly Damaging	NA
**GERP++**	5.92	4.96	NA
**phyloP 100way Vertebrate**	8.904	7.455	NA
**Mutation Taster**	Disease-Causing	Disease-Causing	NA
**FATHMM**	Uncertain	Benign Supporting	NA
**SIFT**	Pathogenic Supporting	Benign Moderate	NA

^a^ Meta Score: These predictors determine a pathogenicity based on the combined evidence from multiple other in silico predictors. Note: Engines are assigned a prediction points score based on the strength of the calibrated prediction. Supporting: 1 point. Moderate: 2 points. Strong: 4 points. Very Strong: 8 points. NA: information not available.

## Data Availability

The exome sequencing data presented in this study will be available through NCBI dbGAP database.
